# AI performance assessment in blended learning: mechanisms and effects on students’ continuous learning motivation

**DOI:** 10.3389/fpsyg.2024.1447680

**Published:** 2024-12-16

**Authors:** Hao Ji, Lingling Suo, Hua Chen

**Affiliations:** Management College, Beijing Union University, Beijing, China

**Keywords:** AI performance assessment, blended learning, continuous learning motivation, expectation confirmation model (ECM), educational technology

## Abstract

**Introduction:**

Blended learning combines the strengths of online and offline teaching and has become a popular approach in higher education. Despite its advantages, maintaining and enhancing students’ continuous learning motivation in this mode remains a significant challenge.

**Methods:**

This study utilizes questionnaire surveys and structural equation modeling to examine the role of AI performance assessment in influencing students’ continuous learning motivation in a blended learning environment.

**Results:**

The results indicate that AI performance assessment positively influences students’ continuous learning motivation indirectly through expectation confirmation, perceived usefulness, and learning satisfaction. However, AI performance assessment alone does not have a direct impact on continuous learning motivation.

**Discussion:**

To address these findings, this study suggests measures to improve the effectiveness of AI performance assessment systems in blended learning. These include providing diverse evaluation metrics, recommending personalized learning paths, offering timely and detailed feedback, fostering teacher-student interactions, improving system quality and usability, and visualizing learning behaviors for better tracking.

## 1 Introduction

With the rapid advancement of information technology, particularly the rise of artificial intelligence (AI), educational technology has gained widespread application. Blended learning combines the advantages of online and offline instruction, offering a more flexible and efficient teaching model for higher education (Yang et al., 2023). However, a persistent challenge in this teaching model is how to continuously stimulate students’ learning motivation (Zhang et al., 2016). Although research indicates that blended learning can enhance students’ learning experiences and engagement, their sustained motivation often remains limited. This limitation arises from the higher demands placed on students’ self-discipline and autonomous learning capabilities, causing some students to feel lost or unmotivated during their studies (Wang and Huang, 2023).

While numerous studies have explored the role of AI in enhancing educational outcomes, few have delved into how AI-enabled assessment systems affect students’ sustained motivation in blended learning environments. Existing research largely focuses on the technical advantages of AI in optimizing teaching processes or providing personalized feedback (Guo et al., 2023; Rad et al., 2023). However, understanding the mechanisms through which these systems operate—particularly how they influence key motivational constructs such as expectation confirmation, perceived usefulness, and learning satisfaction in an integrated teaching model—remains limited. This study addresses this research gap by applying the Expectation Confirmation Model (ECM) and Self-Determination Theory (SDT) to explore how AI performance assessment systems can enhance students’ sustained motivation in blended learning.

The potential theoretical contributions of this study include verifying the impact of AI performance assessment on students learning experiences and expanding the application of the ECM and SDT. By analyzing the influence of AI performance assessment technology in blended learning, this research reveals the critical roles of expectation confirmation and perceived usefulness in enhancing students’ motivation for continuous learning, providing a theoretical foundation for future research in related fields. Practically, this study offers valuable insights for educational institutions and technology developers. The findings suggest that AI performance assessment systems should focus on designing personalized feedback and enhancing perceived usefulness to improve student learning satisfaction and intentions for continuous learning. Effective application of technology in blended learning environments can significantly enhance students learning experiences and provide strong support for long-term motivation.

## 2 Literature review and research hypotheses

**Figure 1:**
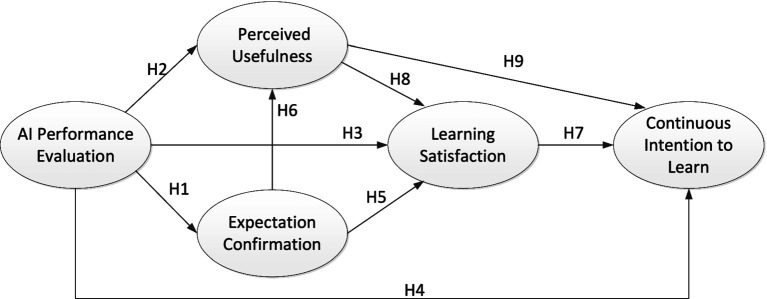
Model framework and assumptions.

Figure 1 presents the conceptual framework of this study, which examines the influence of AI-driven performance assessment on students’ continuous intention to learn within a blended learning environment. This model is grounded in two primary theoretical frameworks: Self-Determination Theory and the Expectation Confirmation Model. These theories underscore the essential psychological and motivational factors that contribute to sustained learning engagement and satisfaction. Within this framework, AI performance assessment functions as an independent variable, influencing three key psychological responses among students: expectation confirmation, perceived usefulness, and learning satisfaction. These responses collectively shape students’ continuous intention to learn.

Specifically, expectation confirmation reflects the extent to which students perceive that their experience with AI assessment aligns with or surpasses their initial expectations. When students’ expectations are met or exceeded, they are more likely to view the assessment as both useful and satisfying. Perceived usefulness, on the other hand, captures students’ beliefs about the value that AI assessment adds to their learning experience and outcomes; higher perceived usefulness is often linked to greater satisfaction and a stronger inclination to continue utilizing AI tools for learning. Learning satisfaction encompasses students’ overall evaluation of their experience with AI-driven assessment, shaped by factors such as personalized feedback, autonomy, and real-time support provided by the AI system. Satisfied students are generally more inclined to engage persistently in the learning model.

This framework posits several causal relationships among these constructs, suggesting that continuous intention to learn is influenced by both satisfaction and perceived usefulness derived from AI performance assessments. With this conceptual foundation in place, the specific hypotheses are developed as follows.

### 2.1 The impact of AI performance assessment on students

SDT posits that individual motivation is driven by three fundamental psychological needs: autonomy, competence, and relatedness (Ryan, 2017). In blended learning environments, AI grading systems enhance students’ sense of control over their learning processes by providing personalized feedback and immediate support, thereby fostering greater engagement and sustained effort in their studies.

Firstly, AI grading systems facilitate a better understanding of students’ learning progress and areas needing improvement through real-time, personalized feedback. This tailored feedback not only boosts students’ confidence but also statistically significantly enhances learning outcomes by assisting them in adjusting their learning strategies (Guo et al., 2023; Rad et al., 2023). Personalized feedback is particularly effective in blended learning contexts, as it allows students to receive direct responses during their self-directed learning, thereby reducing uncertainty and maintaining high levels of participation (Lim et al., 2020). This feedback mechanism is a crucial factor in expectation confirmation, as it helps students verify whether their learning progress aligns with their goals (Wang and Lehman, 2021). When students receive immediate and specific feedback during their learning journey, they are more likely to affirm their expectations and recognize their progress, thus enhancing their motivation for continued learning. Therefore, we propose the following hypothesis:

*H1:* AI Performance Assessment positively influences the level of expectation confirmation among students in blended learning.

Additionally, AI Performance Assessment systems help students identify knowledge gaps and adjust their learning strategies promptly through automated grading and personalized feedback. This immediate and detailed feedback enhances students’ sense of control over their learning progress in blended learning, reducing their anxiety while waiting for responses (Riezebos et al., 2023) and clarifying how blended learning supports their education. SDT emphasizes that greater autonomy in tasks fosters intrinsic motivation. When students recognize that the system can quickly and accurately identify their learning needs and provide effective improvement suggestions, their perceived usefulness statistically significantly increases (Troussas et al., 2023). Furthermore, students gradually realize that the system not only addresses their current learning challenges but also continuously supports their academic progress, leading to overall performance improvements (Pulfrey et al., 2011). Consequently, students’ trust and reliance on the blended learning system increase, further reinforcing their perception of the system’s importance and usefulness in achieving learning objectives. Therefore, we propose the following hypothesis:

*H2:* AI Performance Assessment positively influences students' perceived usefulness in blended learning.

Furthermore, AI Performance Assessment systems can effectively enhance students’ satisfaction with blended learning. Learning satisfaction refers to students’ overall evaluation of their learning experiences and is influenced by various factors, including the timeliness of feedback, fulfillment of learning outcomes, and personalized support (Huo et al., 2018). AI systems utilize intelligent analysis and algorithms to quickly identify students’ learning needs and provide timely, personalized support and suggestions. This prompt feedback not only meets students’ learning requirements but also enhances their learning experience and recognition of learning outcomes (Gong et al., 2021). When students perceive that their learning needs are effectively addressed, their satisfaction statistically significantly increases (Rad et al., 2023). Particularly in blended learning environments, students require more personalized support; the feedback mechanisms provided by AI help them feel a greater sense of attention, further improving overall learning satisfaction (Huo et al., 2018). Therefore, we propose the following hypothesis:

*H3:* AI Performance Assessment positively influences students' satisfaction with blended learning.

Finally, AI Performance Assessment systems can directly enhance students’ willingness to engage in continuous learning. The concept of “willingness” refers to students’ readiness and motivation to pursue ongoing learning opportunities, which is essential for academic success. According to SDT, intrinsic motivation plays a critical role in this process. When students experience a sense of autonomy and control over their learning, their self-efficacy increases significantly (Chen and Hoshower, 2003). AI systems provide immediate feedback and personalized learning path recommendations, helping students gain a clearer understanding of their learning progress. This sense of control boosts students’ confidence and encourages them to adjust their learning strategies based on feedback. When students can timely adapt their strategies and witness their own progress, their self-efficacy statistically significantly improves (Pulfrey et al., 2011), thereby fostering their willingness to continue learning. Research indicates that AI-based feedback mechanisms not only enhance students’ academic performance but also effectively boost their intrinsic motivation (Fidan and Gencel, 2022). Therefore, we propose the following hypothesis:

*H4:* AI Performance Assessment positively influences students' willingness to engage in continuous learning in blended environments.

### 2.2 Expectation confirmation model and blended learning

The ECM was developed by Bhattacherjee based on Oliver (1980) Expectation Confirmation Theory (ECT) (Bhattacherjee, 2001). Bhattacherjee (2001) posited that users’ behavior in using information systems is highly consistent with consumer behavior in purchasing, where the users’ experience with the information system impacts their willingness to continue using it. ECM merges the pre-purchase expectations and post-purchase perceived effects in ECT into a single variable: perceived usefulness. It suggests that the continued use intention of information technology or information systems is influenced by perceived usefulness, expectation confirmation, and satisfaction. Hayashi et al. (2004) noted that the main difference between ECT and ECM is that ECM focuses on constructs after the technology’s use.

In recent years, the ECM has been widely applied in educational technology research, demonstrating its effectiveness in explaining the sustained use of information technology in education. According to the ECM, after using educational technology, students reevaluate the technology’s usefulness by assessing the difference between their actual experience and their expectations, which then influences their satisfaction and their intention to continue using the technology (Limayem and Cheung, 2008; Shin et al., 2011).

Specifically, in a blended learning environment, when students experience high levels of expectation confirmation, they are more likely to perceive the blended learning model as useful. This perceived usefulness encompasses not only students’ recognition of the teaching tools and resources used in blended learning but also their positive evaluation of learning outcomes in this teaching model (Kumar et al., 2021). Therefore, we hypothesize:

*H5:* Students' degree of expectation confirmation positively influences their perceived usefulness in blended learning.

Furthermore, the ECM suggests that when students’ learning experiences meet or exceed their expectations, they exhibit higher learning satisfaction. In blended learning, this satisfaction comes not only from the learning outcomes but also from the learning process and resources (Gong et al., 2021). Satisfaction with the learning resources and teaching process in blended learning will enhance overall students learning experience and their willingness to continue learning. Therefore, we hypothesize:

*H6:* Students' degree of expectation confirmation positively influences their learning satisfaction in blended learning.

According to ECM theory, satisfaction is a key factor influencing the intention to continue using a system. In a blended learning environment, when students are satisfied with the learning process and outcomes, they are more motivated to continue using this teaching model. Students with high satisfaction are not only content with their current learning but also maintain a positive attitude towards future blended learning, thereby enhancing their willingness for continuous learning (Chen et al., 2022). Therefore, we hypothesize:

*H7:* Learning satisfaction positively influences students' willingness for continuous blended learning.

Perceived usefulness is an important factor affecting student satisfaction. For example, studies have shown that course quality and perceived practicality statistically significantly influence college students’ satisfaction and behavioral intentions (Chen et al., 2022). Additionally, research has found that blended learning statistically significantly improves students’ motivation, emotional state, and satisfaction compared to traditional teaching methods (Lozano-Lozano et al., 2020). If students perceive blended learning as beneficial to their learning outcomes, they are more likely to be satisfied with the entire blended learning experience. Therefore, we hypothesize:

*H8:* Students' perceived usefulness of blended learning positively influences their satisfaction with blended learning.

Perceived usefulness not only affects student satisfaction but also directly influences their continuous learning intention. Studies have shown that information quality and self-efficacy are key factors determining student satisfaction and continued learning intentions (Li and Phongsatha, 2022). Research during the pandemic has also shown that information quality and self-efficacy statistically significantly influence the intention to continue using Learning Management Systems (LMS) (Alzahrani and Seth, 2021). These studies suggest that when students find blended learning statistically significantly beneficial to their learning, they are not only satisfied with their current learning experience but also more willing to continue using this learning method. This continuous use intention reflects students’ recognition of their current learning experience and predicts their positive attitude towards future learning. Therefore, we hypothesize:

*H9:* Students' perceived usefulness of blended learning positively influences their continuous learning intention in a blended learning environment.

## 3 Research methodology

### 3.1 Participants

This study targeted undergraduate business students at the university, covering various disciplines such as accounting, finance, marketing, and international trade. The gender distribution among participants was relatively balanced, with approximately 48% male and 52% female. The grade distribution was as follows: 30% first-year students, 25% second-year, 28% third-year, and 17% fourth-year students. In terms of academic performance, 35% of participants were in the top 20% of their class, 50% were at an average level, and 15% were in the lower percentile. Additionally, 32% of participants reported having prior experience with AI technology.

### 3.2 Data collection procedure

The questionnaire was designed and distributed via an online platform (Wenjuanxing). The introduction of the survey included a brief explanation of the study’s purpose, privacy protection measures, and the principle of voluntary participation. It was emphasized that all data would be anonymized and used solely for academic research, and participants could withdraw at any time without affecting their academic performance or other rights. The questionnaire link was shared through WeChat class and course groups, allowing students to complete it online by clicking the link.

To boost participation, the research team sent regular reminders via WeChat class and course groups throughout the two-week survey period, with two additional reminders sent during the final 3 days to encourage students to complete the survey promptly. A total of 282 questionnaires were distributed, and 202 valid responses were collected, yielding a response rate of 76.6%. Invalid or incomplete questionnaires were removed during the data-cleaning process to ensure data quality and reliability.

### 3.3 Scale design

The questionnaire scales used in this study were primarily based on established and validated scales. The AI performance assessment was designed according to the findings of Swiecki et al. (2022) and Aloisi (2023). The perceived usefulness scale was derived from Davis et al. (1989) and Bhattacherjee (2001); the student expectation confirmation scale referenced Bhattacherjee (2001) and Lee (2010); the student satisfaction scale was based on Thong et al. (2006); and the intention to continue mobile learning scale was adapted from Lee (2010).

After the initial design of the questionnaire, we invited three experts in the field of educational technology to review it. They provided valuable feedback on the relevance, wording, and clarity of the items. Based on their suggestions, we revised the wording to ensure that each item accurately reflects its corresponding construct and is easily understood by participants.

To further verify the reliability of the questionnaire, we conducted a pilot study with 30 students who shared similar characteristics with the main sample. The results showed that the Cronbach’s *α* coefficients for all scales exceeded 0.75, indicating good internal consistency. Based on feedback from the pilot study, we made minor adjustments to certain items to enhance the reliability and validity of the scales.

All scales employed a 7-point Likert scale, ranging from “1” (strongly disagree) to “7” (strongly agree). At the beginning of the questionnaire, we provided clear instructions emphasizing voluntary participation and data confidentiality. We also ensured uniformity in the data collection process, with all participants completing the questionnaire under the same conditions to minimize the influence of external factors on the data.

### 3.4 Data analysis and model evaluation

This study employed Structural Equation Modeling (SEM) for data analysis and hypothesis testing. SEM is a multivariate statistical technique that simultaneously addresses measurement and structural models, making it suitable for analyzing complex relationships between latent variables.

To ensure the validity and reliability of the SEM analysis, sample size adequacy is crucial. According to Hair et al. (2019), there should be at least 5 to 10 observations per free parameter. This study included 18 observed variables across 5 latent constructs, requiring a minimum sample size of 90 (18 × 5). With 202 valid questionnaires collected, the sample size exceeds this threshold, meeting SEM requirements and ensuring the stability of parameter estimates.

Data preprocessing and descriptive statistical analyses were conducted using SPSS 22, addressing any missing values and outliers. Subsequently, AMOS 24.0 was utilized for SEM analysis, including Confirmatory Factor Analysis (CFA) and structural model testing.

Reliability was assessed using Cronbach’s *α* coefficient, with values above 0.7 indicating acceptable internal consistency (Taber, 2018). Convergent validity was evaluated through CFA, ensuring factor loadings exceeded 0.5, composite reliability (CR) was above 0.6, and average variance extracted (AVE) surpassed 0.5 (Fornell and Larcker, 1981). Discriminant validity was confirmed following Hair et al. (2013) by comparing constrained and unconstrained models in CFA; significant differences in chi-square values indicated adequate discriminant validity. Model fit was assessed using multiple indices, including chi-square (χ^2^), degrees of freedom (df), χ^2^/df ratio, Root Mean Square Error of Approximation (RMSEA), Comparative Fit Index (CFI), Incremental Fit Index (IFI), and Tucker-Lewis Index (TLI).

By utilizing these methods, the study effectively assessed the reliability and validity of the measurement and structural models, providing a solid statistical foundation for testing the research hypotheses. Detailed analysis results will be reported in the Results section.

## 4 Research results

We follows the two-step analysis method of structural equation modeling, which includes confirmatory factor analysis to measure the validity of the model constructs and path analysis along with significance testing of the structural model.

### 4.1 Confirmatory factor analysis

The reliability and validity of the scales were evaluated using Confirmatory Factor Analysis (CFA), focusing on three key aspects: reliability, convergent validity, and discriminant validity.

Reliability: The Cronbach’s *α* coefficients for each construct ranged from 0.815 to 0.912, exceeding the recommended threshold of 0.7 (Taber, 2018). This indicates a high level of internal consistency among the items within each construct, confirming good reliability.

Convergent Validity: The factor loadings for all items ranged between 0.612 and 0.936, all above the acceptable minimum of 0.5. Composite Reliability (CR) values were between 0.829 and 0.950, exceeding the recommended threshold of 0.6. The Average Variance Extracted (AVE) ranged from 0.551 to 0.865, surpassing the minimum requirement of 0.5 (Fornell and Larcker, 1981). These results confirm that the scales exhibit good convergent validity.

Discriminant Validity: Discriminant validity was assessed using the method proposed by Hair et al. (2013). The chi-square differences between constrained models (where correlations between constructs are fixed at 1) and unconstrained models (where correlations are freely estimated) ranged from 25.6 to 131.0, all of which were significant at *p* < 0.001. This demonstrates strong discriminant validity between the constructs, indicating that each construct is distinct and measures a unique aspect.

These findings collectively suggest that the measurement model has good reliability and validity, making it suitable for subsequent structural model analysis.

### 4.2 Model fit and path coefficient analysis

Model fit measures the degree of consistency between the theoretically estimated model and the actual sample data. A better fit indicates that the expected covariance matrix aligns more closely with the sample matrix, and good fit indices are essential for structural equation modeling (SEM) analysis. The model fit indices used in this study are shown in Table 1. Specifically, the normalized chi-square value (χ^2^/df) is 2.736, which is below the ideal threshold of 5. The CFI, NFI, IFI, and TLI values are 0.941, 0.911, 0.942, and 0.928, respectively, all exceeding the 0.90 benchmark, indicating good model fit. The RMSEA is 0.043, which is less than 0.08, suggesting a high level of model parsimony. Additionally, the other parsimony fit indices—PGFI, PCFI, and PNFI—are all above 0.50, confirming that the model is not overly complex and further supporting its simplicity. Overall, these results indicate a good fit between the model and the data, allowing for further path analysis.

**Table 1:** Model fit indices.

Fit indices	Ideal standards	Model fit
χ^2^/df	≤5	2.736
CFI	≥0.92	0.941
NFI	≥0.90	0.911
IFI	≥0.90	0.942
TLI	≥0.90	0.928
PGFI	≥0.50	0.634
PCFI	≥0.50	0.769
PNFI	≥0.50	0.744
RMSEA	≤0.08	0.043

**Figure 2:**
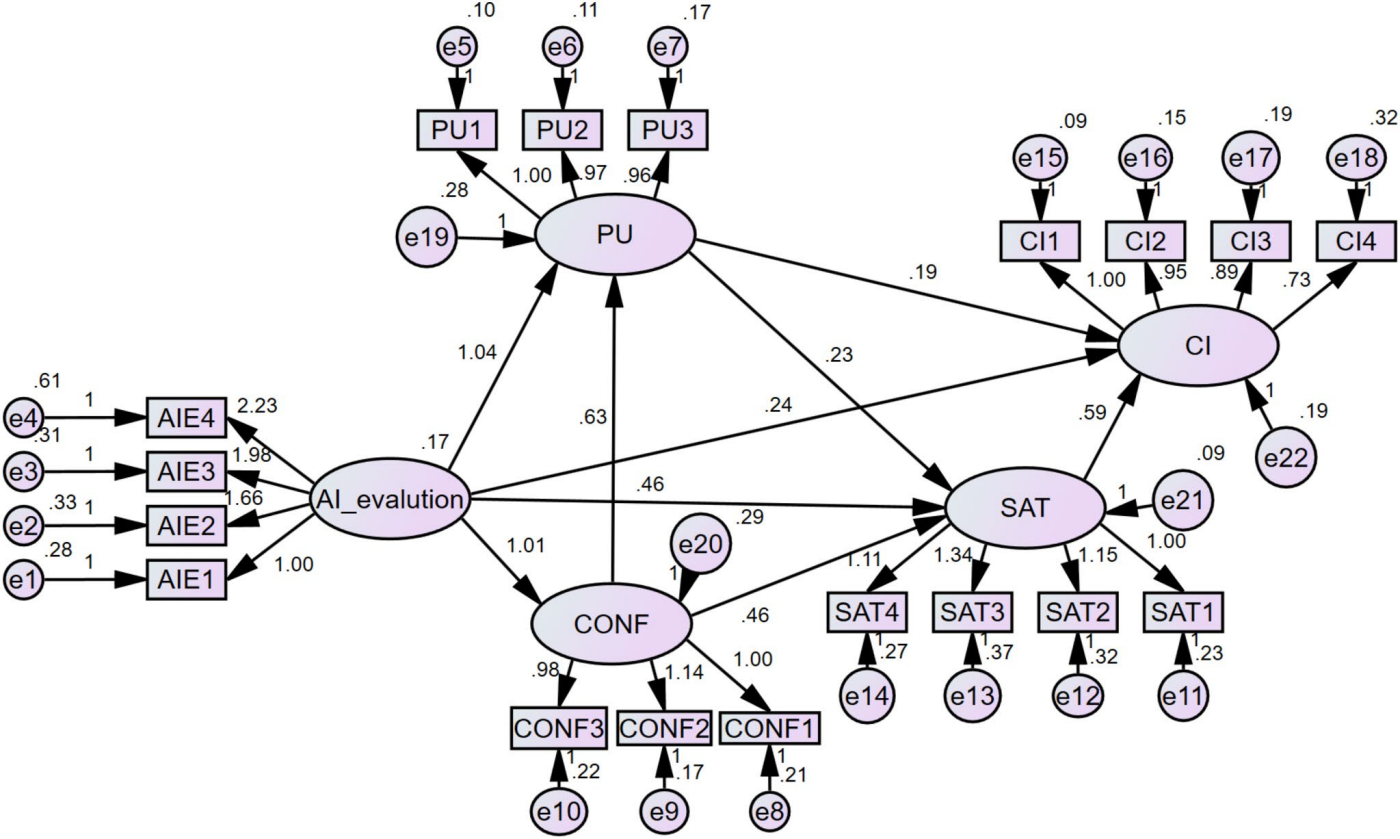
Structural model and path coefficients.

The model fit results are shown in Figure 2, and the standardized path coefficients and hypothesis testing results are presented in Table 2. The analysis indicates that AI performance assessment significantly enhances students’ expectation confirmation (path coefficient = 0.610, *p* < 0.001), supporting hypothesis H1. It also has a significant positive effect on students’ perceived usefulness (path coefficient = 0.455, *p* < 0.001), supporting H2, and significantly improves students’ learning satisfaction (path coefficient = 0.267, *p* < 0.001), supporting H3. However, AI performance assessment does not have a direct significant effect on students’ continuous learning intention (path coefficient = 0.125, *p* = 0.15), which does not support H4.

**Table 2:** Path coefficients and hypothesis testing results.

Path and hypothesis	Standardized coefficient	*T*-value	*p*-value	Conclusion
AI performance assessment→ Expectation Confirmation (H1)	0.61	7.054	***	Supported
AI performance assessment→ Perceived Usefulness (H2)	0.455	6.038	***	Supported
AI performance assessment→ Learning Satisfaction (H3)	0.267	3.571	***	Supported
AI performance assessment→ Intention to Continue Use (H4)	0.125	1.441	0.15	Not Supported
Expectation Confirmation → Perceived Usefulness (H5)	0.461	6.971	***	Supported
Expectation Confirmation → Learning Satisfaction (H6)	0.437	6.027	***	Supported
Learning Satisfaction → Intention to Continue Use (H7)	0.53	5.014	***	Supported
Perceived Usefulness → Learning Satisfaction (H8)	0.303	3.749	***	Supported
Perceived Usefulness → Intention to Continue Use (H9)	0.228	2.395	*	Supported

*, ** and *** denote *p* < 0.05, *p* < 0.01, and *p* < 0.001, respectively.

Further analysis reveals that expectation confirmation has a significant positive effect on perceived usefulness (path coefficient = 0.461, *p* < 0.001), supporting H5, and it also significantly enhances learning satisfaction (path coefficient = 0.437, *p* < 0.001), supporting H6. Learning satisfaction significantly boosts continuous learning intention (path coefficient = 0.530, *p* < 0.001), supporting H7. Perceived usefulness not only has a positive effect on learning satisfaction (path coefficient = 0.303, *p* < 0.001), supporting H8, but also directly increases continuous learning intention (path coefficient = 0.228, *p* < 0.05), supporting H9.

In summary, all hypotheses were supported except H4. The results suggest that AI performance assessment indirectly influences students’ continuous learning intention by enhancing expectation confirmation, perceived usefulness, and learning satisfaction, highlighting the positive role of AI technology in optimizing blended learning.

## 5 Discussion

This study examined the impact of AI performance assessment on students in blended learning environments. The results indicate that all hypotheses, except H4, were supported. These findings align with existing literature and further validate the applicability of SDT and the ECM, emphasizing the critical role of personalized feedback in enhancing the students learning experience. In the context of this study, it is essential to consider the unique cultural and educational environment in which these findings were obtained. Local factors, such as students’ expectations of teacher-student interaction and their familiarity with technology in educational settings, likely influence how AI-based assessments are perceived and utilized. In regions where students highly value close interaction with instructors, AI feedback mechanisms may complement traditional teaching styles by providing continuous support between in-person sessions. Alternatively, in contexts with limited exposure to AI technology, students may require additional guidance to fully engage with these systems.

Firstly, the support for H1 indicates that AI performance assessment significantly improves students’ expectation confirmation. According to Self-Determination Theory (Deci and Ryan, 2013), personalized and timely feedback fulfills students’ basic psychological needs for autonomy and competence, helping them better manage their learning progress, reduce uncertainty, and boost confidence. Through personalized feedback provided by AI technology, students can gain real-time insights into their learning status, clearly identifying which aspects meet their expectations and which require improvement. This process helps students compare their actual learning experiences with their initial expectations, thus strengthening their expectation confirmation. This finding is consistent with Wang and Lehman (2021), who found that personalized feedback not only helps students confirm whether their learning progress aligns with expectations but also enhances their confidence and motivation. In the context of AI performance assessment, precise and customized feedback enables students to promptly adjust their learning strategies, ensuring that their learning experience remains aligned with their expected goals. This alignment enhances students’ expectation confirmation, which contributes to increased learning satisfaction and a stronger intentions to continue participating (Bhattacherjee, 2001). In the local educational context, where students may prioritize clear feedback and guidance, AI-driven feedback fulfills this need by offering real-time, objective evaluations that students might not otherwise receive as consistently. This increased feedback frequency and specificity can play a critical role in meeting students’ expectations, especially in educational systems where personalized feedback from instructors may be limited due to larger class sizes or resource constraints.

The support for H2 demonstrates that AI performance assessment has a significant positive effect on students’ perceived usefulness. Perceived usefulness reflects the sense of competence from SDT, specifically students’ confidence in their ability to master learning tasks and their sense of control over the learning process. Through personalized feedback, AI performance assessment allows students to promptly understand their learning progress, identify strengths and weaknesses, and thus enhance their control over the learning process. This not only boosts students’ recognition of their own learning abilities but also increases their trust and reliance on teaching tools and resources. This finding aligns with the results of Troussas et al. (2023), who found that AI performance assessment enhances students’ trust in the system through precise feedback, thereby increasing their perceived usefulness of the blended learning model. When students feel that they can effectively use these tools to improve their learning outcomes, their perceived usefulness is significantly enhanced, further supporting the positive role of AI performance assessment in blended learning. In regions where students may not have extensive prior experience with technology in educational contexts, the perceived usefulness of AI tools may initially depend on their ease of use and the clarity of feedback provided. By ensuring that AI tools are intuitive and tailored to local educational needs, educators can enhance perceived usefulness and encourage broader acceptance and adoption of AI systems.

To further enhance students’ perceived usefulness, educators should provide timely and targeted feedback through AI performance assessment systems. One effective method is utilizing Learning Analytics Dashboards (LAD) to track and visualize students’ learning behaviors, helping them accurately assess their progress. Additionally, offering a personalized learning experience is key to boosting perceived usefulness. AI systems should recommend relevant supplementary materials and exercises based on students’ learning progress and interests to meet their individual needs and improve learning outcomes. In some educational contexts, these tools could address a gap where students may otherwise lack access to detailed, individualized guidance. By adapting LAD and personalized content to reflect the specific curriculum and cultural expectations, educators can further increase the perceived relevance and value of these tools.

Teacher support and trust are also crucial. To ensure the effective use of AI tools, it is important that these tools do not increase teachers’ workload but instead assist in simplifying the assessment process and providing useful teaching insights. By offering support mechanisms and addressing ethical concerns, educators’ trust and acceptance of AI systems can be strengthened, leading to more positive adoption of these tools in the teaching process and further improving students’ perceived usefulness.

The validation of H3 demonstrates that AI performance assessment significantly improves students’ learning satisfaction. According to SDT, learning satisfaction is derived not only from external feedback but also from a sense of autonomy and control during the learning process. Through personalized feedback provided by AI systems, students gain clearer insights into their learning progress, identify areas that need improvement, and adjust their learning strategies accordingly. This feedback mechanism not only enhances students’ sense of control over their learning tasks but also improves their overall learning experience in blended learning, thereby increasing their learning satisfaction. This finding is consistent with Lim et al. (2020), who found that timely feedback and personalized support play a critical role in improving students’ learning satisfaction. In local contexts where teacher-student relationships are highly valued, the role of AI in supplementing teacher feedback may be especially important. By providing timely and personalized feedback, AI systems can bridge potential gaps in teacher availability, allowing students to feel supported and improving their satisfaction with the learning experience.

To further boost students’ learning satisfaction, educators should fully leverage the advantages of AI systems to ensure that students receive timely and detailed performance feedback. By using AI tools to automatically generate comprehensive evaluations and promptly deliver them to students, educators can help students clearly understand their learning progress and areas for improvement. This not only enhances learning outcomes but also strengthens students’ trust in AI systems. Furthermore, when designing AI systems, it is essential to account for students’ social backgrounds, psychological conditions, and cultural diversity to offer inclusive and personalized features that increase learning satisfaction. For example, in regions with diverse linguistic backgrounds, AI systems that offer multilingual support or culturally relevant content can enhance satisfaction and make the learning process more inclusive. This kind of personalized support effectively meets students’ psychological needs for autonomy, competence, and relatedness, thereby improving their overall learning experience.

Additionally, improving the quality and usability of AI systems is vital. Through regular updates and system optimizations, educational institutions can ensure that students experience convenient and efficient operations while using the system. This reduces technical barriers and enhances students’ positive experience with the system, leading to greater learning satisfaction. To address the diverse needs of students, providing easy-to-understand user guides and necessary training can help students use AI systems more effectively, giving them a greater sense of autonomy and control over their learning. Mastery of the system’s functionality can reduce technical difficulties and uncertainty, further increasing students’ satisfaction with the system during their use.

Although H4 was not supported, indicating that AI performance assessment does not have a direct impact on students’ continuous learning intentions, the effects of expectation confirmation and perceived usefulness on continuous learning intentions remain significant through indirect pathways. This aligns with the conclusions of Chen and Hoshower (2003), suggesting that students rely more on the positive experiences gained during the learning process rather than on the direct influence of AI technology itself on their motivation to continue learning. Autonomy and competence, key elements of motivation in SDT, are reinforced when students receive personalized feedback through AI systems and perceive progress in their learning. This boosts their learning motivation and confidence. However, the findings of H4 also indicate that while AI performance assessment enhances students’ sense of autonomy and competence, it is insufficient to directly influence their continuous learning intentions. Instead, it requires mediation through other variables, such as expectation confirmation or perceived usefulness, to have an indirect effect. This suggests that future research could explore how to further enhance the interactivity and personalized support of AI systems to directly impact students’ continuous learning intentions.

The empirical results show that H5 and H6 were supported, consistent with the predictions of the ECM. When students used the AI performance assessment system, they confirmed the alignment between their learning experience and expectations, which enhanced their perceived usefulness and learning satisfaction. This is consistent with Bhattacherjee (2001) ECM, which states that the higher a user’s expectation confirmation, the more their perceived usefulness and satisfaction increase.

To strengthen the expectation confirmation mechanism, educators should introduce a diverse set of assessment metrics that comprehensively reflect students’ learning progress, ensuring the thoroughness and accuracy of evaluation results. Additionally, integrating AI-based teaching evaluations with intelligent tutoring systems and chatbots can enhance students’ understanding and mastery of the learning process by providing real-time support. A third approach is to adopt a comprehensive assessment method that combines quantitative and qualitative metrics, to ensure the reliability of students’ performance assessments. By leveraging AI to analyze vast amounts of learning data, educators can better understand students’ learning conditions and needs, thereby meeting their expectations.

The results for H7, H8, and H9 demonstrate that learning satisfaction and perceived usefulness have a significant impact on students’ continuous learning intentions. Learning satisfaction not only directly enhances students’ willingness to continue participating in blended learning, but perceived usefulness also influences continuous learning intentions by improving learning satisfaction and through direct effects. This aligns with Davis (1989) Technology Acceptance Model (TAM), which emphasizes the central role of perceived usefulness and satisfaction in technology adoption and continued use. Students’ recognition of teaching tools and resources, as well as the satisfaction they gain from the learning process, are key drivers of sustained learning motivation.

To further enhance learning satisfaction and perceived usefulness, educators and technology developers should implement a variety of strategies. First, optimizing instructional design by providing high-quality content and a user-friendly experience will better meet students’ practical needs. Second, diversifying learning resources to cater to different students’ interests and needs can increase the appeal and engagement of the learning process. Additionally, improving the user interface of AI systems to make them more intuitive and convenient will reduce barriers to usage. Establishing incentive mechanisms, such as points and rewards, can also motivate students to participate more actively, boosting their learning motivation and engagement with the system. Finally, strengthening data security and privacy protection will ensure that students’ data is handled appropriately, enhancing their trust in the system.

In summary, this study reveals the relationships between AI performance assessment, expectation confirmation, perceived usefulness, learning satisfaction, and continuous learning intentions. The validation of multiple pathways enriches the existing theoretical framework, highlighting the importance of meeting students’ psychological needs and enhancing the learning experience in technology-enhanced education. This research also expands the application of the ECM and SDT.

On a practical level, the findings offer valuable insights for educational institutions and technology developers. First, AI performance assessment systems should focus on the design of personalized feedback to enhance students’ sense of autonomy and competence. Second, improving students’ perceived usefulness and satisfaction with teaching tools is key to promoting their continuous learning intentions. Thus, instructional design should be centered on students’ actual needs, offering valuable learning resources and support. Although AI technology itself did not directly increase continuous learning intentions, it can indirectly achieve this goal by improving the students’ learning experiences. This underscores that in blended learning, the effective use of technology should be student-centered, with a focus on enhancing expectation confirmation, perceived usefulness, and learning satisfaction to foster students’ sustained learning motivation.

## 6 Conclusion

This study summarizes the role of AI performance assessment systems in blended learning and their influence on students’ continuous learning intentions. By integrating SDT and the ECM, this study demonstrates the multiple pathways through which AI performance assessment systems affect students’ learning motivation. The findings show that AI performance assessment does not directly impact students’ continuous learning intentions but instead exerts an indirect effect through key mediating variables such as expectation confirmation, perceived usefulness, and learning satisfaction. This discovery broadens the application of existing theories, revealing how technology tools enhance intrinsic motivation by fulfilling students’ psychological needs for autonomy, competence, and relatedness. Expectation confirmation plays a crucial role in this process, enhancing students’ learning experience and outcomes in technology-enhanced learning environments.

On a practical level, the study offers valuable guidance for educators and technology developers. To maximize the positive impact of AI performance assessment on learning outcomes, educational institutions should design AI systems that meet or exceed students’ expectations, thereby improving perceived usefulness and learning satisfaction, which in turn promotes continuous learning motivation. Educators should focus on providing personalized feedback to address students’ learning needs, improving system usability and functionality, and ensuring that these systems fulfill students’ psychological needs for autonomy, competence, and relatedness. Technology developers should prioritize optimizing user experience, protecting data privacy, and providing AI tools that simplify educators’ workloads without adding to their burden.

However, this study has some limitations. First, the data were collected solely from business students at one university. Future research could expand the sample to include students from various disciplines and educational levels to better validate the generalizability of the findings. Second, this study used a survey method for data collection, which may be subject to social desirability bias. Future research could incorporate experimental designs or longitudinal studies to more comprehensively assess the long-term impact of AI performance assessment on students’ learning motivation and performance. Finally, while this study examined the overall effects of AI performance assessment systems, it did not delve into the specific performance of different types of AI tools in various educational contexts. Future studies could analyze the application and potential impact of AI tools such as adaptive learning platforms, virtual teaching assistants, and intelligent feedback systems across different teaching scenarios to better understand the role of AI in education.

## Data Availability

The raw data supporting the conclusions of this article will be made available by the authors, without undue reservation.

## Appendix

**Table A1:** Research variable items.

Latent variable	Item		Statement
AI performance assessment	AIE1	I believe that AI technology can reduce human bias in the evaluation process.	Swiecki et al. (2022) and Aloisi (2023)
	AIE2	I believe that AI technology can provide detailed feedback to help me improve my learning.	
	AIE3	AI technology can promptly provide my learning performance and feedback.	
	AIE4	AI technology is reliable in assessments, and I trust its scoring standards.	
Expectation confirmation	CONF1	The learning experience in blended learning is better than I expected.	Bhattacherjee (2001) and Lee (2010)
	CONF2	The quality of blended learning is better than I expected.	
	CONF3	Most of my expectations for using blended learning were met after use.	
Perceived usefulness	PU1	I can engage in blended learning anytime, anywhere.	Davis (1989) and Bhattacherjee (2001)
	PU2	I find blended learning to be useful.	
	PU3	Blended learning has improved my learning efficiency.	
Learning satisfaction	SAT1	Overall, I am satisfied with the experience of blended learning.	Thong et al. (2006)
	SAT2	I am satisfied with my experience using blended learning.	
	SAT3	I can feel the joy of learning during blended learning.	
	SAT4	I am satisfied with the learning outcomes of blended learning.	
Continuance intention	CI1	I intend to continue using blended learning in the future.	Lee (2010)
	CI2	I will choose more blended learning courses in the future.	
	CI3	I will continue to use blended learning in my future studies.	
	CI4	The learning experience in blended learning is better than I expected.	
